# Data Resource Profile: the Children and Young People with Long COVID (CLoCk) Study

**DOI:** 10.1093/ije/dyad158

**Published:** 2023-11-21

**Authors:** Manjula D Nugawela, Snehal M Pinto Pereira, Natalia K Rojas, Kelsey McOwat, Ruth Simmons, Emma Dalrymple, Tamsin Ford, Shruti Garg, Dougal Hargreaves, Malcolm G Semple, Laila Xu, Roz Shafran, Terence Stephenson, CLoCk Consortium, Marta Buszewicz, Trudie Chalder, Esther Crawley, Bianca De Stavola, Anthony Harnden, Isobel Heyman, Shamez Ladhani, Michael Levin, Vanessa Poustie, Terry Segal, Kishan Sharma, Olivia Swann, Elizabeth Whittaker

**Affiliations:** Population, Policy and Practice Research and Teaching Department, UCL Great Ormond Street Institute of Child Health, University College London, London, UK; Research Department of Targeted Intervention, Division of Surgery & Interventional Science, Faculty of Medical Sciences, University College London, London, UK; Research Department of Targeted Intervention, Division of Surgery & Interventional Science, Faculty of Medical Sciences, University College London, London, UK; Immunisations and Vaccine Preventable Diseases, UK Health Security Agency, London, UK; Immunisations and Vaccine Preventable Diseases, UK Health Security Agency, London, UK; Population, Policy and Practice Research and Teaching Department, UCL Great Ormond Street Institute of Child Health, University College London, London, UK; Department of Psychiatry, University of Cambridge, Hershel Smith Building Cambridge Biomedical Campus, Cambridge, UK; Division of Neuroscience, School of Biological Sciences, University of Manchester, Manchester Academic Health Science Centre, Manchester, UK; Child and Adolescent Mental Health Services, Royal Manchester Children’s Hospital, Manchester Academic Health Sciences Centre, Manchester, UK; Mohn Centre for Children’s Health & Wellbeing, School of Public Health, Imperial College London, London, UK; NIHR Health Protection Research Unit in Emerging and Zoonotic Infections, Institute of Infection, Veterinary and Ecological Sciences, University of Liverpool, Liverpool, UK; Respiratory Medicine, Alder Hey Children’s Hospital, Liverpool, UK; Population, Policy and Practice Research and Teaching Department, UCL Great Ormond Street Institute of Child Health, University College London, London, UK; Population, Policy and Practice Research and Teaching Department, UCL Great Ormond Street Institute of Child Health, University College London, London, UK; Population, Policy and Practice Research and Teaching Department, UCL Great Ormond Street Institute of Child Health, University College London, London, UK

**Keywords:** Long COVID, children and young people, cohort study

Key Features

- The Children and Young People with Long COVID (CLoCk) study is a national matched prospective study which was set up with the aims of (i) describing the clinical phenotype of post-COVID symptomology in children and young people (CYP), (ii) producing a research definition for long COVID in CYP and (iii) establishing the prevalence of long COVID in CYP.
- In total, 219 175 CYP aged 11–17 years, who were tested for SARS-CoV-2 via polymerase chain reaction (PCR) testing between September 2020 and March 2021 in England, were invited to participate. Test-positive and test-negative CYP were matched at study invitation on month of test, age, sex and geographical region.
- CYP were enrolled into the study at 3, 6 or 12 months after their index PCR test (*n* = 31 012). Depending on when they enrolled, they were also invited to fill in follow-up questionnaires at 6, 12 and 24 months after their index test. The overall response rate was 14.1%, with retention across sweeps varying from 36.6% to 54.1%.
- The dataset includes information on physical and mental health using validated scales over time, allowing for examination of within-individual change. CYP report symptoms themselves rather than relying on parental report or administrative records.
- Requests for access to the participant-level data from this study can be submitted via email to: [Clock@ukhsa.gov.uk].

## Data resource basics

### Context and challenges

The Long COVID in Children and Young People (CLoCk) study began in April 2021 against a backdrop of uncertainty surrounding the diagnosis, phenotype, prevalence, duration and treatment of long COVID among children and young people (CYP). At the time, there was limited evidence suggesting that around 10% of people infected with SARS-CoV-2 experience prolonged symptoms after initial infection.^1^ CYP were mostly asymptomatic or had low symptom burden at the time of acute infection compared with adults.^2–4^ However, the longer-term consequences of infection among CYP were unclear, with some CYP reporting ongoing symptoms such as fatigue, dyspnoea, heart palpitations, chest pain, headaches, difficulties in concentrating, muscle weaknesses and sore throat 6 to 8 months after clinical diagnosis of COVID-19.^5^

There was a clear need to identify CYP at higher risk of having longer-term consequences due to COVID-19 infection and the prevalence of long COVID among CYP, while considering the wider impact of the pandemic. During the early days of the pandemic, risk factors found to be associated with long COVID in adults were older age, female sex, obesity, mental health problems and ethnic minority status.^3^^,^^5^ However, it was not clear if these risk factors were replicated among CYP.

In this context, the National Institute for Health and Care Research (NIHR) and UK Research and Innovation (UKRI) funded CLoCk study, the largest national, prospective, matched cohort study of long COVID in CYP in England was set up. The study had three broad aims of (i) describing the clinical phenotype and prevalence of long COVID among test-positive compared with test-negative CYP, (ii) producing an operational research definition of long COVID in CYP and (iii) establishing the prevalence of long COVID in CYP testing positive for SARS-CoV-2 infection. This data resource profile describes the CLoCk study design and the data collected at each sweep and explains how researchers were proactive during the pandemic, adding new questions into the questionnaire as knowledge was accumulated and the pandemic progressed. Thus, this manuscript acts as a central document with information on the CLoCk data structure and the variables ,to aid future researchers using CLoCk data and those wishing for an in-depth understanding of the sample for existing publications.^6–15^

### Data structure

The CLoCk study is based on the SARS-CoV-2 polymerase chain reaction (PCR)-testing dataset held by Public Health England [now UK Health Security Agency (UKHSA)]. PCR testing for SARS-CoV-2 was undertaken by hospital and public health laboratories, with information on tests, regardless of result, reported to UKHSA as part of mandatory reporting. Data reporting through laboratory information management systems (LIMS) include patient identifiable and demographic information and data relevant to the test. Data were enhanced through linking to the Patient Demographic Service (PDS) to exclude any CYP who had died since testing. We selected participants from England aged 11–17 years who had PCR tests between September 2020 and March 2021. During this period, 234 803 CYP tested positive for SARS-CoV-2 and 1 203 996 tested negative. When the study sample was being selected, among those who tested negative, 76 689 CYP were excluded because they had a positive test result before and/or after their negative test. Test-positives were then matched to test-negative CYP based on age (at last birthday) at time of testing, sex, month of test and geographical region (based on lower super output area). All matched individuals were contacted. However, those who tested in December 2020 and were first contacted 6 months after testing were invited at a ratio of 1 (test-positive):2 (test-negative), as the numbers were so large that not all could be contacted by mail due to initial funding constraints. Matched CYP who were uncontactable (i.e. no address available) or were included in a previous study^16^ were not sent an invitation to participate. In total, after these exclusions, 219 175 CYP (91 014 test-positive and 128 161 test-negative) were invited to participate.

As explained in the study protocol,^17^ a letter was mailed to CYP inviting them to participate in the study using an online link. The link provided information about the study, with an option to consent online and complete a short recruitment questionnaire (paper options were also available). CYP were able to request support in completing the questionnaire from a parent, relative, carer or friend if they wished (e.g. if they had a special educational need).

### Recruitment and enrolment

**Figure 1.**
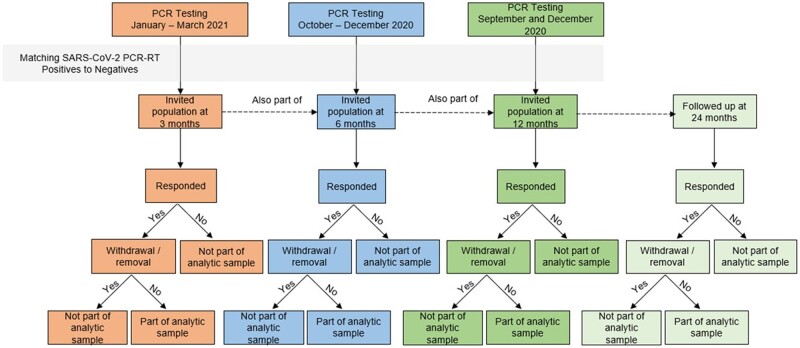
The Children and Young People with Long COVID (CLoCk) study—participant flow from invited to final sample—logic diagram. Adapted from: Rojas NK, De Stavola BL, Norris T, *et al*. Developing survey weights to ensure representativeness in a national, matched cohort study: results from the Children and young people with Long Covid (CLoCk) study. 15 May 2023, preprint (Version 1) available at Research Square [https://doi.org/10.21203/rs.3.rs-2912362/v1]. PCR, polymerase chain reaction; PCR-RT, polymerase chain reaction reverse transcription

Recruitment and enrolment started in April 2021, the exact timing of which depended on the month of PCR testing (see Table 1 and Figure 1). CYP who tested for SARS-CoV-2 in January–March 2021 were invited to enrol into the study in April–June 2021 (i.e. 3 months after testing). CYP who tested in October–December 2020 also enrolled in April–June 2021 (i.e. 6 months after testing). Due to initial funding constraints and the volume of testing in December 2020, only a random sub-set of eligible CYP were invited to participate 6 months after testing in a 1 (test-positive):2 (test-negative) ratio. CYP who tested in September 2020 enrolled into the study in September 2021 (i.e. 12 months after testing).

**Table 1.** The Children and Young People with Long COVID (CLoCk) study survey response rate at each data collection sweep

Month and year of PCR test	Data collection sweeps			
	3 months after testing (responded/invited initially)	6 months after testing (responded/invited initially *OR* responded at first contact at 3 months after testing)	12 months after testing (responded/invited initially *OR* responded at first contact at 3 or 6 months after testing)	24 months after testing (responded/responded at first contact at 3, 6 or 12 months after testing)
September 2020			12.8% (1323/10 387)	38.9% (514/1323)
October 2020		14.2% (3758/26 411)	39.4% (1477/3751)	37.2% (1394/3751)
November 2020		13.2% (5527/41 757)	40.1% (2208/5518)	40.7% (2248/5518)
December 2020		14.0% (1245/8881)	43.5% (539/1239)	39.5% (490/1239)
January 2021	14.6% (5212/35 181)	54.1% (2786/5151)	43.8% (2258/5151)	39.1% (2016/5151)
February 2021	19.4% (1131/5804)	52.0% (584/1124)	41.8% (470/1124)	36.6% (411/1124)
March 2021	10.2% (1013/9860)	48.0% (480/1003)	45.0% (450/1003)	40.5% (406/1002)
December 2020 (additional)^a^			14.6% (11 803/80 894)	43.7% (5153/11 803)

The denominator in each cell represents the number of people invited initially or enrolled into the study; it changes slightly when participants died (*n *=* *1), requested to be withdrawn (*n *=* *9) or did not enrol into the study in a timely manner (e.g. filled in the enrolment and follow-up questionnaires simultaneously) (*n *=* *91).

PCR, polymerase chain reaction.

a December 2020 (additional) refers to the additional participants who were invited to the study at 12 months after testing.

Additional funding was received and the remaining matched CYP who tested in December 2020 (referred to as ‘December additional’) were enrolled into the study in December 2021 (i.e. 12 months after testing). All enrolled CYP were contacted again (depending on enrolment timing) at 6, 12 and 24 months after testing, as described in Table 1 and Figure 1.

A total of 31 012 CYP were enrolled into the study out of 219 175 invited (response rate, 14.1%). CLoCk participants were broadly similar to the invited population, although there were proportionately more females, older CYP and CYP from least deprived areas; there were also some regional disparities (Table 2).

**Table 2.** Characteristics of the invited population and The Children and Young People with Long COVID (CLoCk) study participant population

Characteristic	3 months after testing		6 months after testing		12 and 24 months after testing		
	Invited population (*n* =50 845)	Sample responding at 3 months (*n* =7356)	Invited population (*n* =127 894)	Sample responding at 6 months (*n* =14 380)	Invited population (*n* =219 175)	Sample responding at 12 months (*n* =20 528)	Sample responding at 24 months (*n* =12 632)
Sex							
Female	27 531 (54.2%)	4628 (62.9%)	67 949 (53.1%)	9010 (62.7%)	115 236 (52.6%)	12 762 (62.2%)	8230 (65.2%)
Male	23 314 (45.8%)	2728 (37.1%)	59 945 (46.9%)	5370 (37.3%)	103 939 (47.4%)	7766 (37.8%)	4402 (34.8%)
Age (years)^a^							
11–14	23 340 (45.9%)	3129 (42.5%)	61 589 (48.2%)	6157 (42.8%)	112 057 (51.1%)	10 202 (49.7%)	5760 (45.6%)
15–17	27 505 (54.1%)	4227 (57.5%)	66 305 (51.8%)	8223 (57.2%)	107 118 (48.9%)	10 326 (50.3%)	6872 (54.4%)
Region (England)							
East Midlands	3947 (7.76%)	683 (9.3%)	11 002 (8.6%)	1455 (10.1%)	14 109 (6.4%)	1199 (5.8%)	930 (7.4%)
East of England	7669 (15.1%)	1168 (15.9%)	12 818 (10.0%)	1511 (10.5%)	38 901 (17.8%)	4910 (23.9%)	2549 (20.2%)
London	9768 (19.2%)	1238 (16.8%)	18 128 (14.2%)	1736 (12.1%)	46 300 (21.1%)	4707 (22.9%)	2459 (19.5%)
North East England	1744 (3.4%)	243 (3.3%)	7177 (5.6%)	849 (5.9%)	8613 (3.9%)	601 (2.9%)	455 (3.6%)
North West England	7051 (13.9%)	867 (11.8%)	23 953 (18.7%)	2275 (15.8%)	31 289 (14.3%)	1972 (9.6%)	1382 (10.9%)
South East England	7758 (15.3%)	1208 (16.4%)	15 739 (12.3%)	1921 (13.4%)	31 567 (14.4%)	3559 (17.3%)	2128 (16.8%)
South West England	2792 (5.5%)	562 (7.6%)	6947 (5.4%)	1055 (7.3%)	8139 (3.7%)	780 (3.8%)	647 (5.1%)
West Midlands	6268 (12.3%)	880 (12.0%)	17 134 (13.4%)	1876 (13.1%)	22 681 (10.4%)	1675 (8.2%)	1171 (9.2%)
Yorkshire and the Humber	3848 (7.6%)	507 (6.9%)	14 996 (11.7%)	1702 (11.8%)	17 576 (8.0%)	1125 (5.4%)	911 (7.2%)
IMD quintile							
1 (most deprived)	14 829 (29.2%)	1514 (20.6%)	38 081 (29.8%)	2909 (20.2%)	54 079 (24.7%)	2964 (14.4%)	2026 (16.0%)
2	11 497 (22.6%)	1462 (19.9%)	26 263 (20.5%)	2597 (18.1%)	44 757 (20.4%)	3579 (17.4%)	2217 (17.6%)
3	9137 (17.9%)	1415 (19.2%)	22 135 (17.3%)	2604 (18.1%)	39 876 (18.1%)	3884 (18.9%)	2358 (18.7%)
4	8118 (15.9%)	1419 (19.3%)	21 175 (16.6%)	2982 (20.7%)	39 996 (18.3%)	4612 (22.5%)	2704 (21.4%)
5 (least deprived)	7264 (14.3%)	1546 (21.0%)	20 240 (15.8%)	3288 (22.9%)	40 467 (18.5%)	5489 (26.7%)	3327 (26.3%)

IMD, Index of Multiple Deprivation.

a Age was dichotomised as 11–14/15–17 years, as done in previous CLoCk papers (e.g. Stephenson *et al*., 2022^18^) on the basis of key education stages.

### Patient and public involvement

Patient and public involvement (PPI) meetings took place quarterly. There were 17 CYP who were initially recruited from the study or a precursor study to be part of the PPI team.^16^ There was a core group of 12 active members contributing to the study in multiple ways, including with the Delphi research definition of long COVID (see^18^^,^^19^).

## Data collected

Data collection started at enrolment (in April 2021) and ended in May 2023. The questionnaire was designed to include elements of the International Severe Acute Respiratory and emerging Infection Consortium (ISARIC) Paediatric COVID-19 questionnaire^20^ plus the recent Mental Health of Children and Young People survey.^21^ The enrolment questionnaire included questions on demographics (e.g. ethnicity, height, weight, number of siblings). The next section focused on health and wellbeing ‘just before the COVID-19 pandemic in early March 2020’. It included questions on having an Educational Health and Care Plan (EHCP) in place at school, various health problems (e.g. asthma, allergies, feeling depressed) and a rating of the participant’s physical and mental health using a five-point Likert scale (from very poor to very good). Questions were then asked ‘about your COVID-19 test’, including the number of positive tests they had, the reason for testing and the symptoms at testing. The above-described questions were asked at enrolment only. All questions described below were asked at enrolment and repeated at follow-up, with some exceptions (see below and Table 3).

**Table 3.** Summary of questionnaire data available from the Children and Young People with Long COVID (CLoCk) study

Questionnaire data	At time of testing (retrospectively reported at enrolment)	3 months after testing	6 months after testing	12 months after testing	24 months after testing
Demographics					
Age	x				
Sex	x				
Ethnicity	x				
Height^a^	x	x	x	x	x
Weight now^a^		x	x	x	x
Weight before COVID-19 test^a^	x				
Postcode	x				
Number of siblings^a^	x				
Health and wellbeing before COVID-19					
History of health conditions:					
Asthma	x				
Lung disease other than asthma	x				
Allergy problems (eczema, hay fever, food allergies)	x				
Problems with your stomach, gut, liver, kidneys or digestion	x				
Any neurological disease	x				
Any physical disability	x				
Learning difficulties at school	x				
Educational Care and Health Plan (ECHP)	x				
Problems with sleep	x				
Problems with eating	x				
Were you experiencing:					
A loss of interest or pleasure in doing things	x				
Feeling down, depressed or hopeless	x				
Worrying a lot about bad things or the future	x				
Problems with headaches	x				
Problems with friendships	x				
Feeling tired often	x				
Any other serious ill health	x				
Other questions on:					
Smoking	x				
Use of e-cigarettes	x				
Self-rated physical health	x				
Self-rated mental health	x				
History of medication	x				
History of mental health support	x				
About COVID-19 testing					
Have you had a positive test?	x				
How many positive tests?	x				
The date of the first positive test	x				
If more than one: the most recent positive test date^a^	x				
Have you had a COVID-19 test since the last time you completed this questionnaire?			x	x	x
How many COVID-19 tests have you had?			x	x	x
When was your COVID-19 test (if more than 1: most recent)			x	x	x
What was the result?^a^			x	x	x
If positive: is this your first positive test?^a^			x	x	x
Even if test negative: do you believe you had COVID? (in relation to most recent COVID-19 test)		x	x	x	x
Reason for most recent test (symptoms, near someone who tested positive, school testing, other)		x	x	x	x
When did you first notice symptoms?		x	x	x	x
How long did they last?		x	x	x	x
How bad were the symptoms at their worst?		x	x	x	x
Number of school days missed due to symptoms in the past 4 weeks		x	x	x	x
Symptoms at testing and at follow-up					
Fever	x	x	x	x	x
Chills or shivers	x	x	x	x	x
Persistent cough	x	x	x	x	x
Unusual fatigue/tiredness	x	x	x	x	x
Unusual shortness of breath	x	x	x	x	x
Loss of smell/taste	x	x	x	x	x
Unusually hoarse voice	x	x	x	x	x
Unusual chest pain or tightness in your chest	x	x	x	x	x
Unusual abdominal pain	x	x	x	x	x
Diarrhoea	x	x	x	x	x
Headache	x	x	x	x	x
Confusion, disorientation, or drowsiness	x	x	x	x	x
Unusual eye soreness or discomfort	x	x	x	x	x
Skipping meals	x	x	x	x	x
Dizziness or light-headedness	x	x	x	x	x
Sore throat	x	x	x	x	x
Unusual strong muscle pains	x	x	x	x	x
Earache or ringing in your ears	x	x	x	x	x
Raised, red, itchy welts on the skin or sudden swelling of the face or lips	x	x	x	x	x
Red/purple sores or blisters on feet, including toes	x	x	x	x	x
Problems with your sleep, including getting to sleep, waking in the night or waking early				x	x
Other	x	x	x	x	x
What were your main symptoms (of the symptoms above)?	x	x	x	x	x
How severe would you rate your symptoms? (0–100 scale)^#^				x	x
How much do your symptoms affect your functioning? (0–100 scale)^#^				x	x
GP consultation and hospital admission	x	x	x	x	x
Did your parent talk to doctor about your symptoms?^a^		x	x	x	x
Did you go to the hospital?^a^		x	x	x	x
Did you have to stay overnight?^a^		x	x	x	x
Vaccination					
Have you had vaccination against COVID-19?^a^		x	x	x	x
How many vaccines have you had?^a^^,^^b^				x	x
Which vaccine did you have? (if more than one, refer to first) ^a^^,^^b^				x	x
When did you have the vaccine? ^a^^,^^b^				x	x
What was the second vaccine? ^a^^,^^b^				x	x
When did you have the second vaccine? ^a^^,^^b^				x	x
What was the third vaccine? ^a^^,^^b^				x	x
When did you have the third vaccine? ^a^^,^^b^				x	x
Health at the time of survey					
If you had symptoms, do you agree you have fully recovered (scale 0–10)^a^		x	x	x	x
How do you feel right now?		x	x	x	x
Since the start of your COVID-19 symptoms, have you had a period longer than 1 week with none of the symptoms mentioned above at all?		x	x	x	x
COVID-19 and your family (your house and extended family)					
Has anyone tested positive for COVID-19?^a^		x	x	x	x
Has anyone been to hospital with COVID-19?^a^		x	x	x	x
Has anyone been in intensive care (ICU) with COVID-19?^a^		x	x	x	x
Has anyone died from COVID-19?^a^		x	x	x	x
Does anyone have ongoing problems from COVID-19?^a^		x	x	x	x
Other health-related measures					
Overall health (scale 0–100)	x	x	x	x	x
EQ-5D-Y^c^	x	x	x	x	x
UCLA Loneliness scale	x	x	x	x	x
SDQ		x	x	x	x
Chalder Fatigue Scale		x	x	x	x
SWEMWBS		x	x	x	x
Free text question regarding CYP’s health, the pandemic or lockdown		x	x	x	x

CYP, children and young people; SDQ: Strength and Difficulties Questionnaire; SWEMWBS, Short Warwick-Edinburgh Mental Well-being Scale; UCLA, University of California, Los Angeles.

a Optional questions.

b Questions on symptom severity, symptom impact and detailed vaccination questions were added at 12 months after testing only for CYP who tested in January–March 2021 (i.e. these questions were asked of everyone at 24 months after testing, but only a subset at 12 months after testing).

c EQ-5D-Y is an instrument that evaluates the generic quality of life; EQ-5D-Y is the child-friendly EQ-5D version by the EuroQol Group.

The questionnaire asked ‘about your health at the moment’ and included questions on symptoms experienced ‘right now’ and validated scales including the Strengths and Difficulties Questionnaire,^22^ Chalder Fatigue Scale,^23^ questions on quality of life using EQ-5D-Y,^24^ Short Warwick Edinburgh Mental Wellbeing Scale^25^ and a modified version of the UCLA Loneliness Scale for Children.^26^ We also collected a single direct measure of loneliness (recommended by the Office for National Statistics).^27^ There was a section on ‘COVID-19 and your family’, where we asked about infection and ongoing COVID-19 problems in family members. The final section allowed a free-text response to the question: ‘Please use this space if there is there anything else you would like to tell us about your health or how the pandemic or lockdown have affected you.’. The questionnaires at follow-up were largely unchanged. However, as knowledge accumulated and the pandemic progressed, we added in additional questions: (i) an additional symptom, ‘problems with your sleep, including getting to sleep, waking in the night or waking early’, was added to the list of symptoms asked about at 12 months after testing; (ii) additional questions were added part way through the 12-month post-testing data collection sweep on symptom severity and impact, and questions on type of COVID-19 vaccination received and the date of each dose; and (iii) an amendment to the routing of a school absence question, which was initially asked only of those who stated the reason for their most recent COVID-19 test was that they had symptoms: this was changed in August 2021 so all participants could answer the school absence question [‘In the last four weeks, how many school days (online or in person) in total did you miss because of symptoms of COVID-19?’].

## Data resource use

CLoCk data have generated several important findings on symptom profiles and long COVID in CYP. For example, CLoCk data were used to support developing a Delphi consensus research definition for long COVID in CYP.^18^ This definition was developed and refined in conjunction with participants with lived experience and CYP from the CLoCk PPI group.^18^ It is similar to the recent clinical case definition published by the World Health Organization.^28^ Five studies describe symptom profiles 3, 6 and 12 months after testing, cross-sectionally^6–8^ and longitudinally.^9^^,^^10^ Common symptoms in both test-positive and test-negative CYP included shortness of breath and tiredness. However, test-positive CYP consistently had higher symptom prevalences; for example, 10.9% of test-positive CYP reported tiredness at time of testing, and 6 and 12 months after testing, whereas 1.2% of test-negative CYP reported tiredness at all three time points.^10^ Older (vs younger) CYP and females (vs males) were more likely to develop long COVID.^12^ We also observed an increased risk of long COVID among CYP having a parent/carer with ongoing problems after COVID-19, compared with CYP who did not report parents/carers having ongoing problems from COVID-19.^11^ One further study shows that during the pandemic, females, older adolescents and those from deprived areas were at higher risk of loneliness.^13^ CLoCk data have also shown that there has been a small decline in mental health among participating CYP during the pandemic, with females and older adolescents having a higher risk of deterioration.^29^ In initial CLoCk study publications, test-positive CYP were compared with laboratory-confirmed SARS-CoV-2 test-negative CYP^6^^,^^7^^,^^9^^,^^10^^,^^12^ [and we excluded CYP who had been (re-)infected after their baseline PCR-test]. However, as the pandemic progressed and almost all CYP had SARS-CoV-2 antibodies by mid-2022,^30^ more recent publications take into account both PCR testing information held at UKHSA and self-report of testing. We now consider four groups of CYP: ‘initial test-negatives with no subsequent positive test’; ‘initial test-negatives with a subsequent positive test’; ‘initial test-positives with no report of subsequent re-infection’; and ‘initial test-positives with subsequent report of re-infection’.^8^ Publications in preparation include developing a prediction model for experiencing ongoing symptoms at 12 and 24 months after testing in the initial test-positives only.

## Strengths and weaknesses

A key strength of the CLoCk study is its prospective follow-up of test-positive and test-negative CYP who were matched at baseline on age, sex and geographical area. Moreover, the CLoCk questionnaire gathers information on physical and mental health using the same measures and validated scales over time, allowing us to assess within-individual change. CYP report symptoms themselves rather than relying on parental report or administrative records. However, study limitations are also acknowledged. The CLoCK cohort was drawn from periods when the dominant UK virus strain was the original wild-type SARS-COV-2 (September–December 2020) and the Alpha (B.1.1.7) variant (January–March 2021). Thus, findings from the CLoCk study might not apply to subsequent variants (e.g. Delta, Omicron). We have recently published an additional (smaller) study assessing symptomology after infection with the Omicron variant.^31^ The CLoCk study had a response rate of 14.1% (31 012/219 175), with slightly higher proportions of female and older adolescents and least deprived CYP being more likely to respond compared with those invited. There was little difference in demographic characteristics between participating test-positive and test-negative CYP, reflecting the matched-cohort study design. We have developed flexible survey weights that can be used in all future CLoCk data analyses.^14^ Initial results indicate that published findings^10^ are similar to results when re-weighted to be nationally representative of CYP in England.^14^ It is possible that some CYP might have been misdiagnosed as SARS-CoV-2 negative and vice versa: false negatives might be attributable to the timing of the PCR, swab technique and assay sensitivity, but false-positive PCR results are rare. Despite our more recent publications being responsive to the background rates of infection in England,^8^ we recognize misclassification might still occur. The study design may involve selection bias, for example by favouring those with internet access, and CYP may be more likely to participate if they had symptoms to report. Relatedly, we were specifically funded to study non-hospitalized CYP (i.e. the milder end of the acute COVID-19 spectrum) and over the 2-year study period the proportion of CYP who visited hospital (620/31 012) or stayed overnight (330/31 012) in relation to COVID-19 was relatively low. Thus, whereas our results are likely to be relevant to many COVID-19 cases in CYP, there are undoubtedly some CYP severely affected by chronic debilitating long-term symptoms and our findings may not be generalizable to sub-groups who were hospitalized or seeking treatment in clinics or hospitals.^15^ Finally, baseline data collection was retrospective and therefore prone to recall bias and, although subsequent data collection sweeps were prospective, we cannot infer if or how symptoms varied in the intervening period.

Studying a disease when the background infection rates are changing and knowledge is concurrently accumulating is difficult. It is thus near impossible to have a gold-standard study design and methodology to determine causality. Hence, multiple studies from different settings are required. For example, whereas in the CLoCk study CYP reported on their health, findings can be used in combination with research using administrative data to generate a broader understanding of the impact of COVID-19 on CYP.^8^^,^^32–35^ For this reason, we encourage collaborations where CLoCk data can be used in further studies and in comparisons using other data sources available on long COVID in CYP (see details below).

## Data resource access

The CLoCk study has a website [https://www.ucl.ac.uk/child-health/research/population-policy-and-practice-research-and-teaching-department/champp/psychological-8] providing updates and communicating findings to the public. A signed data access agreement with the CLoCk team is currently required before accessing shared data. All requests for data will be reviewed by the CLoCk study team, to verify whether the request is subject to any intellectual property or confidentiality obligations. Requests for access to the participant-level data from this study can be submitted via e-mail to [Clock@ukhsa.gov.uk] with detailed proposals for approval. We plan on providing open access to the dataset in the future, for example, via the UK Data Archive.

## CLoCK Consortium

Additional co-applicants on the grant application and CLoCk Consortium members (alphabetical): Marta Buszewicz, Trudie Chalder, Esther Crawley, Bianca De Stavola, Anthony Harnden, Isobel Heyman, Shamez Ladhani, Michael Levin, Vanessa Poustie, Terry Segal, Kishan Sharma (sadly deceased), Olivia Swann, Elizabeth Whittaker.

## Ethics approval

Ethics approval was granted by Yorkshire and The Humber—South Yorkshire Research Ethics Committee (REC reference: 21/YH/0060; IRAS project ID: 293495).

## Data Availability

See Data resource access.
